# Methyl Sulfone Induces Loss of Metastatic Properties and Reemergence of Normal Phenotypes in a Metastatic Cloudman S-91 (M3) Murine Melanoma Cell Line

**DOI:** 10.1371/journal.pone.0011788

**Published:** 2010-08-04

**Authors:** Joan McIntyre Caron, Marissa Bannon, Lindsay Rosshirt, Jessica Luis, Luke Monteagudo, John M. Caron, Gerson Marc Sternstein

**Affiliations:** Department of Cell Biology, School of Medicine, University of Connecticut Health Center, Farmington, Connecticut, United States of America; Bauer Research Foundation, United States of America

## Abstract

**Background:**

The most deadly form of cancer is not lung or colon, breast or prostate; it is any cancer that has become metastatic. Mortality due to metastatic melanoma, one of the most aggressive and deadly cancers, has increased steadily over the last several decades. Unfortunately, the arsenal of chemotherapeutic agents available today is most often unsuccessful at extending and improving the life expectancy of afflicted individuals. We sought to identify an effective and nontoxic agent against metastatic melanoma.

**Methodology/Principal Findings:**

We chose to study Cloudman S-91 mouse melanoma cells (sub-clone M3, CCL53.1) because these cells are highly aggressive and metastatic, representing one of the deadliest types of cancer. Melanoma cells also had an experimental advantage because their morphology, which is easily monitored, relates to the physiology of metastatic cells and normal melanocytes. We chose to test methyl sulfone as a chemotherapeutic agent for two reasons. Because of its chemical structure, we speculated a potential anti-cancer activity by targeting microtubules. Equally important, methyl sulfone has a well-established safety profile in humans. Surprisingly, we found that malignant melanoma cells exposed to methyl sulfone demonstrated the loss of phenotypes characteristic of malignant cells, and the reemergence of phenotypes characteristic of healthy melanocytes. Briefly, over time methyl sulfone induced contact inhibition, loss of ability to migrate through an extracellular matrix, loss of anchorage-independent growth, proper wound healing followed by contact inhibition, irreversible senescence followed by arborization with melanosomes in arbors as seen in normal melanocytes.

**Conclusions/Significance:**

Methyl sulfone may have clinical potential as a non-toxic agent effective against metastatic melanoma. Additionally, methyl sulfone has promise as a tool to explore molecular mechanisms of metastatic transformation as well as fundamental processes such as cell migration, contact inhibition, wound healing and cellular senescence.

## Introduction

Undoubtedly, the most serious concern with today's chemotherapeutics is that these agents can be highly effective against primary tumors, but they are virtually ineffective when the disease becomes metastatic. Left untreated, most types of cancer cells in primary tumors will eventually become mobile and invasive to nearby blood or lymph supplies, and metastatic disease will develop. At this stage, treatment most often becomes palliative.

A second concern with current chemotherapy is the narrow differential between doses that kill malignant cells and doses that are toxic to healthy cells. With some chemotherapeutic agents, this differential can be as narrow as two-fold. In practice, clinicians must determine optimal dosage for each patient based on this fine line between benefit (killing cancer cells) and risk (severity of toxicity). Within certain groups of patients, chemotherapy-induced toxicity can be severe and life threatening. For example, elderly patients, patients with co-morbid conditions and patients with metastatic disease may not be able to tolerate the toxic side effects associated with optimal doses. However, a less than optimal dose or a longer period of time between treatments can lead to a poor prognosis. If adverse effects become life threatening, treatment with the causative agent is postponed or more likely terminated.

Clearly, an ideal chemotherapeutic drug would be a non-toxic compound that cures metastatic cancer. To identify compounds that at least come close to this ideal, we searched for two criteria based on a yeast model for drug discovery [1]: first, the compound is non-toxic to healthy cells and, second, the chemical structure of the compound suggests anti-cancer activity.

Methyl sulfone is a naturally occurring small molecule found in all mammals, including humans [2]. Humans do not synthesize methyl sulfone. Rather, methyl sulfone is acquired through diet. A primary source of methyl sulfone for people in the United States is cow's milk [3]. Cows acquire methyl sulfone by eating grasses. One type of grass, the plant genus *Equisetum* (horsetail), is found throughout the grasslands of North America and contains high levels of methyl sulfone. Potential toxicity of methyl sulfone has been extensively tested [4], [5]. For example, because methyl sulfone is used as a solvent in manufacturing, toxicity studies of methyl sulfone were performed to determine safety levels for workers who would be exposed to the compound. These studies concluded that methyl sulfone is essentially non-toxic to humans.

The chemical structure of methyl sulfone, C_2_H_6_SO_2_, is almost identical to a known microtubule-binding compound, dimethyl sulfoxide or DMSO (C_2_H_6_SO). Thirty or more years ago, DMSO (10–15%) was a promising new anti-cancer drug because it induced polymerization and stabilization of microtubules [6], which in turn it was believed, would inhibit mitosis and kill proliferating cancer cells. However, extensive studies of DMSO showed no anti-cancer properties [7], [8]. In fact, DMSO became an organic solvent for other chemotherapeutic drugs such as Taxol [9], [10].

Nevertheless, we explored the possibility that the small differences in the chemical structures and properties of DMSO and methyl sulfone may be sufficient for methyl sulfone to be an effective anti-cancer drug. We show here that methyl sulfone rendered metastatic melanoma cells from the Cloudman M3 cell line harmless by permanently reverting their metastatic phenotypes into what appeared to be non-malignant phenotypes of healthy melanocytes. The clinical significance is that methyl sulfone may be a non-toxic chemotherapeutic drug that is effective against metastatic melanoma.

## Materials and Methods

### Materials

Methyl sulfone and dimethyl sulfoxide were purchased from Fluka/Sigma Co., St. Louis, MI. Alexa Fluor 488-conjugated phalloidin was purchased from Invitrogen, Inc., Eugene, OR.

### Cell Culture

Cloudman S-91 mouse melanoma cells (sub-clone M-3, CCL 53.1; American Type Culture Collection, Rockville, MD), referred to here as Cloudman M3 melanoma cells, were grown in RPMI supplemented with 7% fetal bovine serum (Invitrogen, Inc). Cultures were passaged twice a week.

### Live Cell Microscopy

Cells were plated on 35 mm tissue culture dishes at a concentration of 10^5^ cells/plate, unless stated otherwise, and incubated at 37°C, 5% CO_2_. Cells were videotaped with a Nikon TE300 inverted microscope equipped with a 10×0.25 NA Plan Achromat objective lens. Time series (10 min) of phase contrast images were acquired at a video rate of 1 frame/5s with a Watec-902B CCD video camera (Watec Corp., Japan) via the stream acquisition option of Metamorph image acquisition and analysis software (Universal Imaging Corp., Downington, PA). During recordings, cells were kept at 37°C with 10 mM Hepes, pH 7.4. Time series of cells with and without methyl sulfone were obtained at 0–120 minutes after adding the compound and every 24–48 hours for up to eight weeks.

### Fixed Cell Microscopy of Actin Filaments

Cells were plated on 35mm tissue culture dishes at a concentration of 10^5^ cells/plate and incubated at 37°C, 5% CO_2_. After 24 hours, medium was replaced with control medium (RPMI without methyl sulfone) or RPMI with 200 mM methyl sulfone. After melanoma cells reached confluence, cells were fixed in 4% paraformaldehyde and actin filaments were stained with phalloidin-Alexa-488. Cells were imaged with a Perkin Elmer Ultraview RS5 spinning-disk confocal scanning system mounted on a Nikon TE2000 inverted microscope with a 63×1.4 NA Plan Apo oil immersion objective. (William A. Mohler, Ph.D., Director, Spinning Disk Microscope Facility, University of Connecticut Health Center).

### Cell Proliferation

Cells (10^5^ cells/35 mm plate) were plated in RPMI. After 24 hours, medium was replaced with RPMI containing 0, 50 mM, 100 mM, 200 mM and 600 mM methyl sulfone. At several time points, cells were released from plates by trypsinization and counted with a hemocytometer. Apoptosis was assessed with the Annexin V-FITC Apoptosis Kit (PharMingen; Becton-Dickinson, San Diego, CA). Images were obtained at the Richard D. Berlin Center for Cell Analysis and Modeling, University of Connecticut Health Center, Farmington, CT, with an Axioplan CCD Microscope equipped with a 40×1.3 NA FL objective lens, equipped with a Photometrics PXL-EEV37 high speed digital cooled CCD camera via Metamorph image acquisition and analysis software (Universal Imaging Corp., Downington, PA). All assays were performed at least three times and in triplicate.

### DNA Synthesis

DNA synthesis was assayed by incubating cells with bromodeoxyuridine (BrdU) followed by fixation and incubation with Alexa Fluor 488-conjugated monoclonal anti-BrdU antibody as described by the manufacturer (Molecular Probes, Eugene, OR). Images were obtained at the Richard D. Berlin Center for Cell Analysis and Modeling, University of Connecticut Health Center, Farmington, CT, with an Axioplan CCD Microscope equipped with a 40×1.3 NA FL objective lens, equipped with a Photometrics PXL-EEV37 high speed digital cooled CCD camera via Metamorph image acquisition and analysis software (Universal Imaging Corp., Downington, PA).

### Cell Invasion

Invasion assays were performed using Transwell Chambers with 8 µm pores (Corning, Inc., Lowell, MA). Membranes were coated with extracellular matrix gel (Engelbreth-Holm-Swarm murine sarcoma, Sigma Co., MI) that was diluted 1∶6 with RPMI with and without 200 mM methyl sulfone. Membranes were placed in chambers containing RPMI with and without 200 mM methyl sulfone. Cells (1x10^5^ cells) were seeded into upper chambers in RPMI with and without 200 mM methyl sulfone. After 48 and 72 hours at 37 °C, 5% CO_2_, the number of cells that migrated through the ECM was determined using an Axioplan CCD microscope equipped with a 40×1.3 NA FL objective lens and high speed digital cooled CCD camera via Metamorph image acquisition and analysis software (Universal Imaging Corp., Downington, PA). All assays were performed at least three times and in triplicate.

### Soft Agar Colony Formation

Cells from stock plates (RPMI with no methyl sulfone) were detached from plates by trypsinization and counted. Cells (5x10^3^ cells) were gently suspended in 37°C RPMI containing 0.66% agar (DNA grade; Difco Bacto Agar; Becton Dickenson and Company, MD) with and without 200 mM methyl sulfone. The suspension was placed on solidified 1% agar in RPMI with and without 200 mM methyl sulfone. Cells were incubated at 37°C with 5% CO_2_. After 14 days, cells were stained with crystal violet. Each 35 mm plate was photographed and colonies were counted with a dissecting microscope. All visible colonies were counted. All assays were performed at least three times and in triplicate.

### Cell Wounding

Cells were plated on 35 mm tissue culture dishes in RPMI. When cultures were confluent, medium was replaced with RPMI with and without 200 mM methyl sulfone. After 48 hours cells were wounded with a sterile plastic 1000 µl pipette tip. Cells were washed twice with medium to remove cell debris and incubated at 37°C, 5% CO_2_ in RPMI with and without 200 mM methyl sulfone. Wound edges were photographed and video taped with a Nikon TE300 inverted microscope equipped with a 10×0.25 NA Plan Achromat objective lens. Time-series (5min) of phase contrast images were acquired at a video rate of 1 frame/3 s with a Watec-902B CCD video camera (Watec Corp., Japan) via stream acquisition option of Metamorph image acquisition and analysis software (Universal Imaging Corp., Downington, PA). During recordings, cells were kept at 37°C with 10 mM Hepes, pH 7.4. Time series and photographs of cells with and without 200 mM methyl sulfone were obtained every 24 hours for up to 120 hours.

### Senescence Assay

Senescence was determined with the Senescence Cells Histochemical Staining Kit (Sigma Co, MI) as described by the manufacturer. From 10 random fields, total number of cells and number of cells stained blue with β-galactosidase were counted after photographing images with a Nikon TE300 inverted microscope equipped with a 10×0.25 NA Plan Achromat objective lens and an Olympus IM microscope with a 20x objective and a Nikon L2 digital camera. All assays were performed at least three times and in triplicate.

## Results

### Methyl Sulfone Induced Contact Inhibition in Metastatic Melanoma Cells

**Figure 1:**
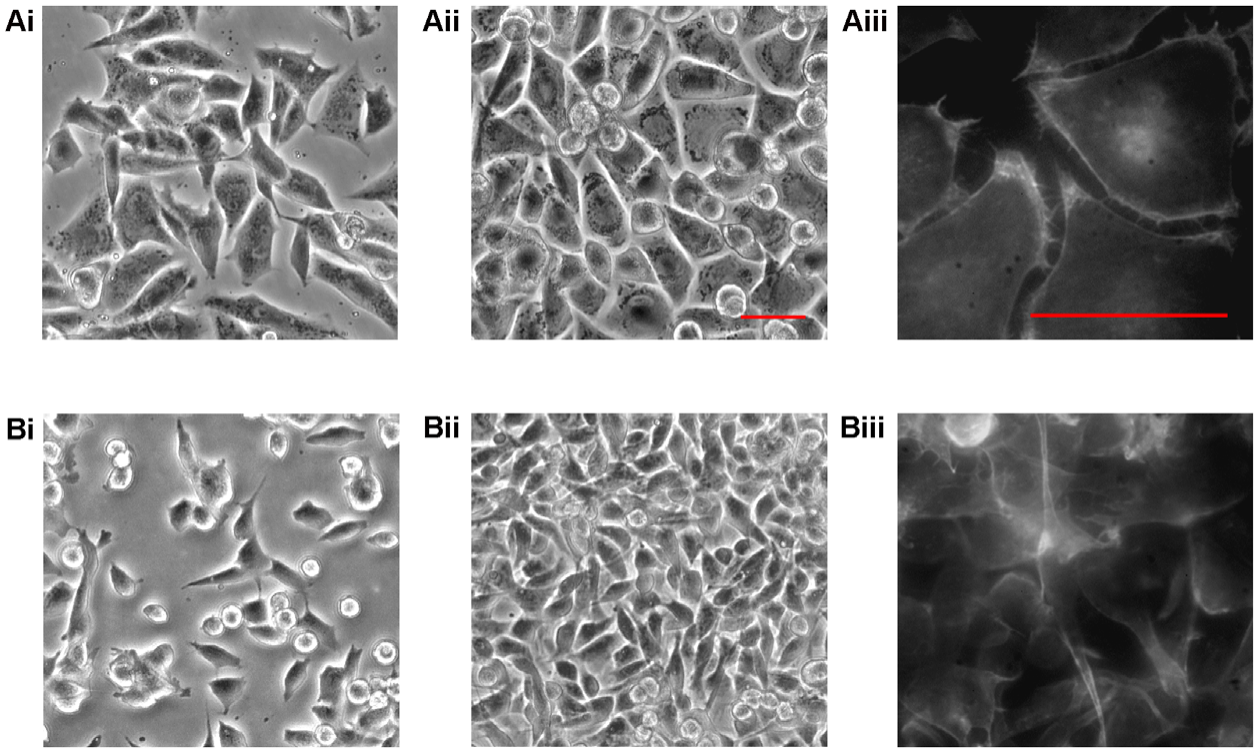
Methyl sulfone induced contact inhibition in metastatic melanoma cells. Melanoma cells (10^5^ cells/ml) were plated into RPMI medium. After 24 hours, medium was replaced with RPMI with and without 200 mM methyl sulfone. (A) Melanoma cells in 200 mM methyl sulfone. Live cell images were acquired 24 hours after media changes (1Ai; Pre-confluent) and every 24 hours for 4 days (1Aii; Confluent); (1Aiii) fixed cell microscopy of Alexa Fluor 488-conjugated phalloidin-stained actin filaments in confluent methyl sulfone-cells; Movie S1: confluent methyl sulfone-treated cells. (B) Melanoma cells without methyl sulfone (Control). Live cell images were acquired 24 hours after media changes (1Bi; Pre-confluent) and every 24 hours for 4 days (1Bii; Confluent); (1Biii) fixed cell microscopy of Alexa Fluor 488-conjugated phalloidin-stained actin filaments in confluent control cells; Movie S2: confluent control cells. Scale bars correspond to 40 µm.

Live cell video microscopy demonstrated that metastatic melanoma cells in 200 mM methyl sulfone reproduced and migrated until cells came in touch with neighboring cells. At this point, cells in methyl sulfone stopped migration and formed a confluent monolayer of contact inhibited cells (**Figure 1A** **i, 1Aii; Movie S1**). Consistent with contact inhibition is the presence of actin filaments localized in small, finger-like protrusions along the plasma membrane as shown in methyl sulfone-treated cells (**Figure 1A** **iii**). Live cell microscopy showed that these actin-filled protrusions were constantly “tapping” neighboring cells across the small space between contact inhibited cells. In contrast, melanoma cells cultured in the absence of methyl sulfone (control) retained an amorphous worm-like shape, and after reaching confluence, continued to migrate under and over neighboring cells (**Figure 1B** **i, 1Bii; Movie S2**). In addition, actin filaments in melanoma cells in the absence of methyl sulfone (control cells) were not localized to plasma membrane protrusions; instead, actin filaments displayed a more chaotic appearance throughout the cytoplasm (**Figure 1B** **iii**).

After treating melanoma cells with 0, 200 mM and 400 mM methyl sulfone for 20 hours, approximately 22% of cells at 0–400 mM methyl sulfone underwent apoptosis (p>0.99) indicating no statistical difference in the percent of apoptotic melanoma cells cultured in media with 0–400 mM methyl sulfone. At 600 mM, methyl sulfone induced apoptosis in 63% of the cells (p<0.05 as assessed by Chi-Square analysis) demonstrating statistical significance. Induction of apoptosis is the outcome most often sought for identifying effective anti-cancer compounds. However, we decided to focus our studies on 200 mM methyl sulfone first, because initial data demonstrated the melanoma cells remained viable at 200 mM and this gave us the opportunity to decipher the actions of this compound. Second, surprisingly our initial experiments demonstrated that 200 mM methyl sulfone induced contact inhibition and therefore might prevent progression of the disease by inducing more normal phenotypes and fewer metastatic phenotypes in melanoma cells.

Methyl sulfone is structurally related to dimethyl sulfoxide (DMSO), and is in fact a metabolite of DMSO [5]. To determine whether DMSO induced a similar morphology as methyl sulfone, we replaced 200 mM methyl sulfone with an equimolar concentration of DMSO. Microscopic analysis demonstrated that DMSO had no apparent effect on the melanoma cells after at least one week in culture.

We next examined the possibility that methyl sulfone caused a change in extracellular osmolarity. We compared melanoma cells in control medium, 200 mM methyl sulfone and 200 mM urea. We chose urea because it has a chemical structure and dipole moment that is similar to methyl sulfone. However, urea alone did not mimic the effects of methyl sulfone. Instead, within approximately two hours, urea induced more then 95% of the cells into necrotic cell death. Finally, methyl sulfone (50–1000 mM) did not alter pH of the medium.

### Methyl Sulfone Inhibited Cell Proliferation and DNA Synthesis in Melanoma Cells

**Figure 2:**
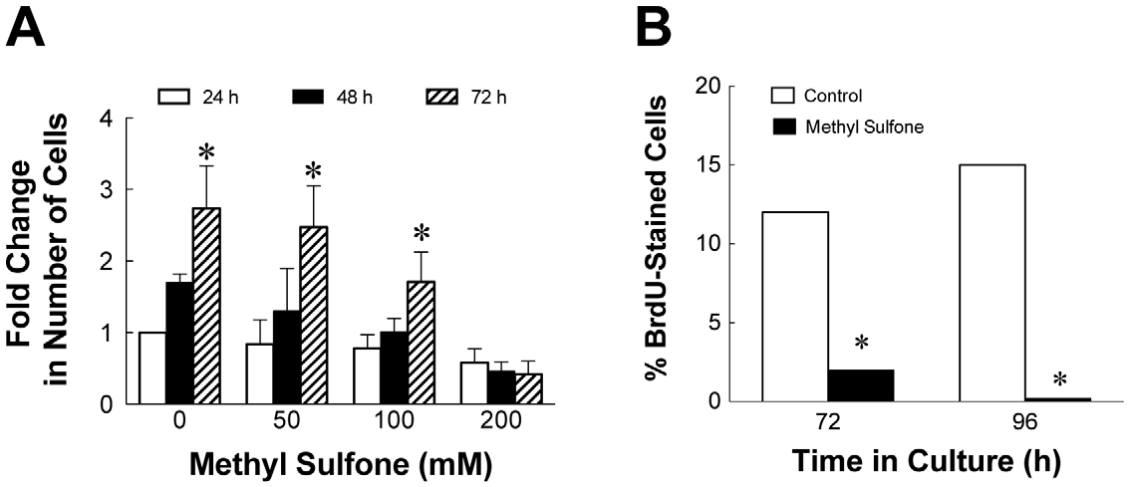
Effect of methyl sulfone on cell proliferation and DNA synthesis. (A) Cell Proliferation: metastatic melanoma cells were incubated with 0, 50 mM, 100 mM and 200 mM methyl sulfone for up to 72 hours. At each time point, cells were trypsinized from plates and counted. (B) DNA Synthesis: melanoma cells were incubated with and without 200 mM methyl sulfone for up to 96 hours. DNA synthesis was measured by incorporation of bromodeoxyuridine (BrdU) followed by fixation and incubation with Alexa Fluor 488-conjugated monoclonal anti-BrdU antibody. Data presented in (A) and (B) are means +/− SEM. Asterisks indicate significance between groups (p value = 0.0001). Data shown in Figure 2A was initially analyzed using a two-way analysis of variance, which revealed a significant effect of both dose and time of treatment. Subsequently these data were analyzed by a one-way analysis of variance followed by a Dunnett's post hoc test to detected differences between different doses and times of exposure. Data in Figure 2B was assessed by Chi-Square analysis at both the 72 and 96 hour time points.

Contact inhibition is associated with G1 cell cycle arrest, also referred to as a state of quiescence. When cells become quiescent, cell proliferation and DNA synthesis is significantly reduced. To determine the effect of methyl sulfone on cell proliferation, melanoma cells were incubated with methyl sulfone (0–200 mM). At 24, 48 and 72 hours, cells were counted as described in methods. As shown in **Figure 2A**, cell proliferation was significantly inhibited in the presence of 200 mM methyl sulfone (p<0.0001).

To compare DNA synthesis in control cells and cells treated with 200 mM methyl sulfone, we examined incorporation of bromodeoxyuridine (BrdU) into newly synthesized DNA. **Figure 2B** shows data from cells at 72 and 96 hours in the presence or absence of 200 mM methyl sulfone. At 72 hours, 14% of control cells were synthesizing DNA while only 0.03% of cells in 200 mM methyl sulfone were synthesizing DNA. At 96 hours, 21% of control cells were synthesizing DNA. In contrast, we found no evidence of DNA synthesis in cells incubated in methyl sulfone for 96 hours (p<0.0001).

### Methyl Sulfone Inhibited Migration of Melanoma Cells through an Extracellular Matrix

**Figure 3:**
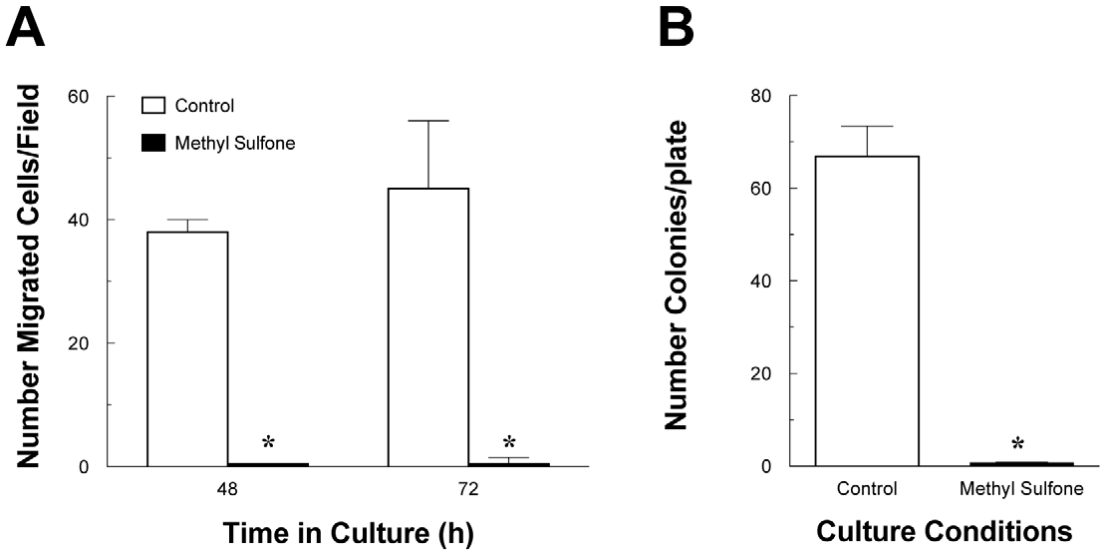
Effect of methyl sulfone on migration of metastatic melanoma cells through an extracellular matrix (ECM) and on anchorage-independent growth. (A) Migration through ECM: metastatic melanoma cells in the absence of methyl sulfone migrated through the extracellular matrix as expected. In contrast, melanoma cells in 200 mM methyl sulfone did not migrate through the ECM. (B) Anchorage-independent Growth: in the absence of methyl sulfone, melanoma cells were able to grow and form colonies on soft agar. In contrast, no colonies were apparent when melanoma cells were in the presence of 200 mM methyl sulfone. Data presented in (A) and (B) are means +/− SEM. Asterisks indicate significance between groups (p value = 0.0001). Data shown in Figure 3A and B were analyzed by a Student's “t” test. All the statistical analysis was performed using Prism 5.0 (GraphPad Software, La Jolla, CA) and differences between groups were considered to significant if p<0.05.

A classic characteristic of metastatic cells is their ability to migrate through an extracellular matrix. As reviewed by Sahai in 2007 [11], metastatic cells utilize at least two types of mechanisms for migration that are essentially not associated with mature normal cells: first, expression of matrix metalloproteinases breaks down the extracellular matrix allowing metastatic cancer cells to tunnel through tissue; second, metastatic cells utilize their amorphous shapes and amoeboid movements to literally squeeze through small spaces (diameters of approximately 5 µm). Clinically, migration away from the primary tumor relates to invasion of metastatic cells into surrounding and then distal tissues. We determined whether methyl sulfone altered this ability in melanoma cells. In the absence of methyl sulfone, more than 200 cells migrated through the matrix. In contrast, none of the cells treated with 200 mM methyl sulfone migrated through the matrix **(** **Figure 3A**; p<0.0001).

### Melanoma Cells Became Anchorage-dependent in the Presence of Methyl Sulfone

Malignant cells do not require attachment or anchorage to a substratum for growth; instead cancer cells will proliferate and form colonies when seeded onto culture plates containing soft agar. This anchorage-independence is another classic characteristic of melanoma cells. In contrast, normal cells will not proliferate and therefore will not form colonies on soft agar. We tested whether methyl sulfone affected anchorage-independent growth of melanoma cells using the soft agar assay. As shown in **Figure 3B**, no colonies formed when melanoma cells were incubated with 200 mM methyl sulfone while more than 300 colonies formed in the absence of methyl sulfone (p<0.0001).

### Wound Healing Proceeded Normally in the Presence of Methyl Sulfone

**Figure 4:**
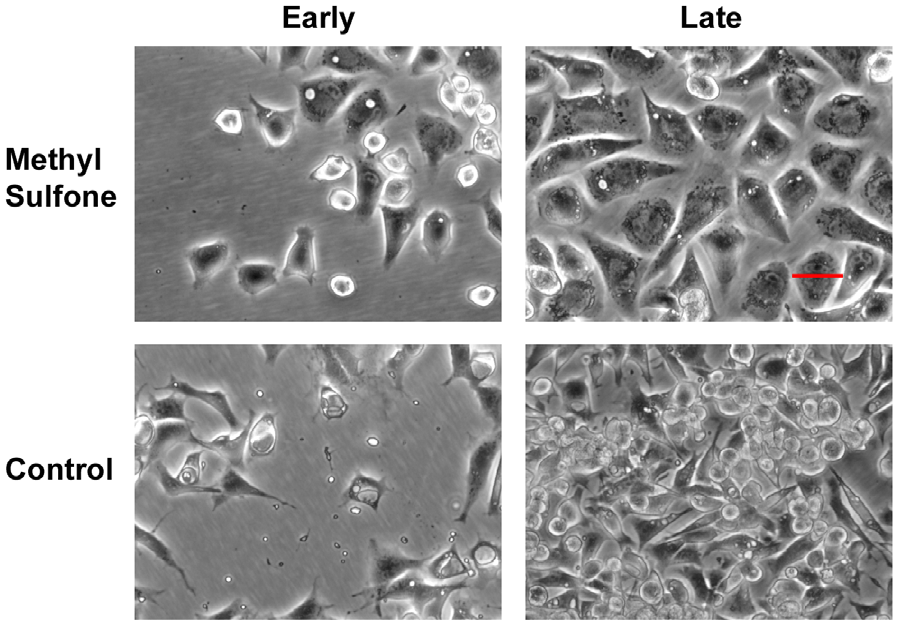
Effect of methyl sulfone on wound healing. Melanoma cells were grown to confluence in the presence and absence of 200 mM methyl sulfone. Scraping a sterile plastic pipette tip through the layer of cells generated a wound. Live cell microscopy was used to monitor wound healing. Shown are cells photographed at 24 hours after wounding (Early) and three days after wounding (Late). Melanoma cells in the presence and absence of 200 mM methyl sulfone were able to migrate into the wounds as expected for proper healing. However, once the wound was covered or healed, cells in 200 mM methyl sulfone stopped migration and once again became contact inhibited. In contrast, melanoma cells in the absence of methyl sulfone did not properly heal the wound; instead these cells continued to grow and move once the wound was covered. Scale bars correspond to 40 µm.

Wound healing is a complicated process in which contact inhibited cells juxtaposed to a wound site must detach from a substratum, migrate into the wounded area and then become contact inhibited once the wound is covered. We tested whether melanoma cells treated with 200 mM methyl sulfone would function properly in the process of wound healing. Melanoma cells in the presence and absence of 200 mM methyl sulfone were grown to confluence. Scraping a layer of confluent cells with a sterile plastic pipette tip formed a wound. Using live cell microscopy we monitored migration of cells into wounds. Melanoma cells treated with 200 mM methyl sulfone migrated into the wound area. When the wound was covered, cells treated with 200 mM methyl sulfone stopped migrating and once again became a confluent monolayer of contact inhibited cells (**Figure 4**). In control samples, cells migrated into the wound area, but did not stop migrating once the wound site was covered, forming a tumor-like mass at the wound site.

### Over Time Methyl Sulfone Induced Senescence in Melanoma Cells

**Figure 5:**
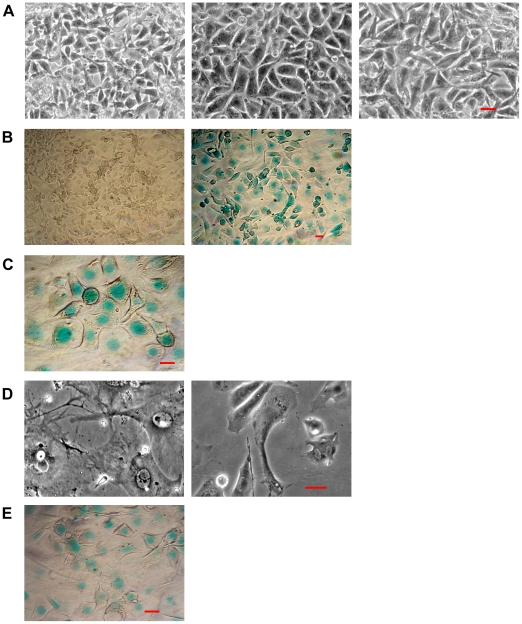
Over time, methyl sulfone induced senescence in metastatic melanoma cells. (A) During the first week of incubation, media was switched at 24-hour intervals between medium containing no drug (control) and medium with 200 mM methyl sulfone. Shown are three photographs of the same plate of cells taken at consecutive 24-hour intervals for three days. The left frame shows melanoma cells in control medium after the first 24-hour interval. The middle frame shows the same plate of cells in 200 mM methyl sulfone after the second 24-hour interval. The right frame shows the cells back in control medium after the third 24-hour interval. At each change of media, melanoma cells cycled between no contact inhibition in control medium to contact inhibition in the presence of methyl sulfone. (B) After incubation for 2–3 weeks in the presence of 200 mM methyl sulfone, metastatic melanoma cells became senescent as shown by blue staining of cells due to increased β-galactosidase activity; control cells continued to migrate and proliferate displaying virtually no blue coloring. (C) To determine if β-galactosidase activity truly indicated the cell cycle arrest that accompanies senescence, melanoma cells were incubated in medium containing 200 mM methyl sulfone. After three weeks, the methyl sulfone medium was replaced with control medium. Cells were incubated for an additional two weeks in drug-free medium and then tested for β-galactosidase activity. The cells remained senescent. (D) To further test the validity of methyl sulfone-induced senescence, melanoma cells were incubated in medium with and without 200 mM methyl sulfone for three weeks. Cells were then trypsinized and both control cells and methyl sulfone cells were re-plated in medium without methyl sulfone. After incubation for an additional two weeks, melanoma cells that were initially grown in methyl sulfone once again became contact inhibited while control cells showed no signs of contact inhibition. (E) Melanoma cells were incubated in 200 mM methyl sulfone. After three weeks, cells were trypsinized and replated into drug-free medium. After two weeks, the presence of senescent cells was demonstrated by β-galactosidase activity. Scale bars correspond to 40 µm.

During the first two weeks of incubating melanoma cells in 200 mM methyl sulfone, replacement of the methyl sulfone medium with drug-free medium resulted in the reappearance of metastatic phenotypes (**Figure 5A**). In other words, within 24 hours of incubation in drug-free medium, cells lost contact inhibition and began proliferating and migrating. At this point we replaced the drug-free medium with medium containing 200 mM methyl sulfone. Once again, the cells became contact inhibited. This cycle of reversal stopped when the cells were incubated in 200 mM methyl sulfone for more than two weeks. As shown in **Figure 5B**, after three weeks in the presence of methyl sulfone, greater than 99% of the melanoma cells became senescent as judged by β-galactosidase activity which turns senescent cells blue [12], [13]. In contrast, less than 0.1% of control cells were senescent.

Senescence indicates that cells can never re-enter the cell cycle. To determine whether the increase in β-galactosidase activity truly indicated senescence, we replaced medium containing 200 mM methyl sulfone with drug-free medium on cells that the β-galactosidase assay indicated were senescent. At one, two and three weeks after replacing methyl sulfone medium with drug-free medium, we found that the cells remained senescent (**Figure 5C**). As a further test, we detached methyl sulfone-induced senescent cells from the culture dish by trypsinization and replated the cells in medium without methyl sulfone. Within 24 hours of replating, methyl sulfone-primed cells reattached to tissue culture plates in medium without methyl sulfone, and became contact inhibited; in contrast, control cells replated into medium without methyl sulfone displayed no contact inhibition (**Figure 5D**). Finally, methyl sulfone-treated cells that were replated into medium without methyl sulfone again displayed β-galactosidase activity (**Figure 5E**).

### Methyl Sulfone Induced Arborization of Senescent Melanoma Cells

Mature melanocytes assume a morphology that is similar to neuronal cells by having a small area of cytoplasm surrounding the nucleus and long extensions called arbors. The primary function of melanocytes is to produce melanin and package the melanin in vesicles called melanosomes. Melanosomes are then transported to the outermost region of arbors. The arbor tips are phagocytized by keratinocytes, cells that sit near the skin's surface, and the newly acquired melanosomes form an umbrella-like shield around the nuclei of keratinocytes to protect the DNA of these cells from UV-induced mutations.

**Figure 6:**
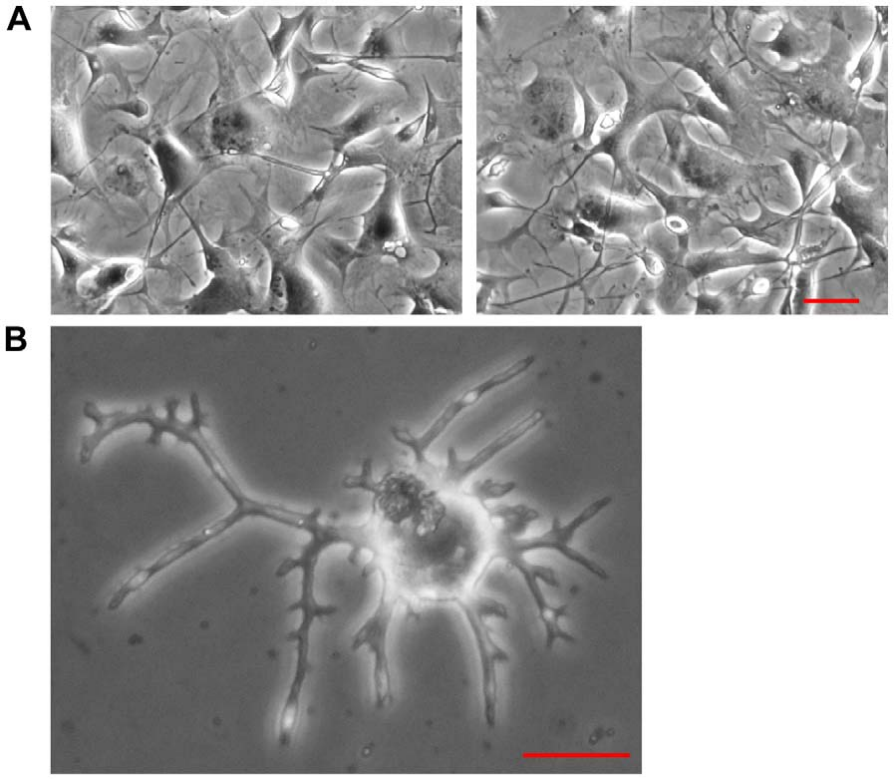
Once senescent, methyl sulfone induced the arborization of metastatic melanoma cells. (A) Examples of arborized melanoma cells. Cells were photographed and videotaped with a Nikon TE300 inverted microscope equipped with a 10×0.25 NA Plan Achromat objective lens. (B) A highly arborized melanoma cell that was incubated in 200 mM methyl sulfone for four weeks. Cells were photographed using an Olympus IM microscope with a 20x objective and a Nikon L2 digital camera. Scale bars correspond to 40 µm.

Surprisingly, we found that virtually all of methyl sulfone-induced senescent melanoma cells took on morphology similar to mature melanocytes complete with extensive arbors that were filled with melanosomes (**Figure 6**). These methyl sulfone-induced senescent and arborized cells remained viable in culture for at least three months in the presence or absence of methyl sulfone.

## Discussion

### Malignant or Metastatic Melanoma

We wanted to test methyl sulfone against the most difficult cancers to cure. Therefore, we focused our study on metastatic disease because primary tumors of most cancers are curable while metastatic cancer is rarely curable. We chose metastatic melanoma because this is one of the most aggressive and deadly cancers. Unlike most cancers, melanoma can occur at any age, from children to the elderly. Additionally, the incidence of melanoma is rapidly increasing worldwide. Over the last 30 years the incidence of malignant melanoma increased by 50% among women aged 15–39. While early stage melanoma has a five-year survival rate of 92–97%, late stage or metastatic melanoma has a five-year survival rate of 15–20% [14].

Melanoma cells have an experimental advantage because their morphology, which is easily monitored, relates to the physiology of both metastatic cells and normal melanocytes. For example, after migrating from the neural crest to the epidermis, the morphology of melanocytes changes from an amorphous worm-like shape that is hydrodynamically ideal for migration to an arborized shape similar to neuronal cells that is optimal for production and transfer of melanin-containing vesicles, melanosomes, to keratinocytes.

### Methyl Sulfone

Methyl sulfone is a small (94.33 mol wt), water-soluble compound that humans obtain from specific food sources such as cow's milk and a variety of vegetables [3]. Interestingly, vegetables that contain methyl sulfone have been identified as possible anti-cancer agents, but for compounds other than methyl sulfone; examples include broccoli, cabbage and Brussels sprouts. Over the last 50 years, the level of methyl sulfone has decreased in foods we eat [15]. This decrease is due, at least in part, to an increase in food processing including pasteurization [3]. It is premature to speculate on any causal connections between reduction of methyl sulfone in our environment and the increased incidence of cancer, but the possibility is interesting. We know that industrialization has introduced cancer-causing compounds into our environment. But the opposite, putting an anti-cancer compound back into our environment, would be progressive.

### Methyl Sulfone and Lack of Toxicity

We realize that some people will think that the concentration of methyl sulfone (200 mM; approximately 2%) used in our studies seems high and that this concentration may not be obtainable in human subjects, and if obtainable this concentration would likely be toxic to patients. In our *in vitro* studies, we show that Cloudman M3 melanoma cells in 200 mM methyl sulfone are alive for months and appear to be healthy arborized melanocytes. However, testing of methyl sulfone for toxicity was well in progress by the 1960's.

For example, male and female rats were given a daily dose of methyl sulfone for 90 days at a concentration of approximately 15% at a rate of 1.5 g/kg per day. [5]. This concentration is approximately 750 percent higher than the concentration we used in our *in vitro* studies. Based on the method of administration (gavage), we felt that the stomach and duodenum would be the most likely areas to find abnormalities. Animals were checked for clinical signs of toxicity (e.g., body weight; food consumption) and mortality twice a day. No signs of toxicity were observed. Laboratory tests were performed before initiation of the experiment and at week seven. These included blood tests: red blood cells, white blood cells, platelets, hematocrit, mean corpuscular hemoglobin, differential leukocyte count, blood coagulation, prothrombin time, liver enzymes, lipid profile, serum protein, albumin, serum sodium, serum potassium; urinalysis: appearance, volume, specific gravity, pH, protein, glucose, blood. At 90 days, animals were fasted for 16 hours and then euthanized. A full gross necropsy included weighing and examining liver, kidneys, adrenals, testicles, spleen, brain, thymus, heart, mesenteric lymph nodes, submandibular lymph nodes, stomach, duodenum, pancreas, lungs, pituitary, trachea, esophagus, thyroids, parathyroids, epididymis, prostate, uterus and ovaries. Finally, a bone marrow smear was performed on all animals. Analysis of these data showed no adverse effects on any of the parameters listed above for both males and females.

### Methyl Sulfone and Contact Inhibition

Contact inhibition, first identified by Kruse and Miedema [16] and Stoker and Rubin [17], is a complicated process that is still not well understood [18]. However, it is clear that contact inhibition is critical for developmental organization of multicellular organisms and for reorganization of tissue after injury [19]. We show here that Cloudman M3 melanoma cells in the presence of methyl sulfone remain contact inhibited until injury or wounding. At this point, Cloudman M3 melanoma cells in methyl sulfone undergo the proper process of wound healing: directed migration into the wound and becoming contact inhibited once the wound is covered. In contrast, control cells migrate and cover the wound, but show no contact inhibition; instead, control cells do not stop once the wound is healed, producing a tumor-like mass at the wound site.

We show here that in the presence of methyl sulfone, Cloudman M3 melanoma cells produce a confluent monolayer of flattened contact inhibited cells. At this early stage of contact inhibition, we noticed that a small number of spherical-shaped cells appeared to be tethered to areas of the tissue plate that were not covered with flattened contact inhibited cells. In fact, we saw no evidence that these round cells ever spread out or grew over the contact inhibited cells. Live cell microscopy demonstrated that these rounded cells were not dead. Nevertheless, we examined these spherical cells by gently pipeting the cells off of the culture dish (under these conditions, contact inhibited cells in the confluent monolayer did not detach) and the spherical cells were plated into fresh tissue plates in RPMI with 200 mM methyl sulfone. Interestingly, these once spherical cells now became flattened and contact inhibited. These data suggest that methyl sulfone-induced contact inhibition was strong enough to prevent the spherical cells from growing on top of the original flattened and contact inhibited cells. We propose that these spherical cells were “moored” to a region of the tissue culture plate that was not covered with contact inhibited cells. However, if space does develop, such as in wounding, the spherical cells will flatten and become contact inhibited.

### Methyl Sulfone and the Instability of Lethal Metastatic Phenotypes

Studies presented here demonstrate that the fundamental nature of metastatic melanoma cells can be reversed. This suggests that methyl sulfone may have the potential to override the effects of cancer-related changes and induce the reemergence of normal melanocyte phenotypes. This apparent ability of methyl sulfone to induce differentiated phenotypes in metastatic Cloudman M3 melanoma cells puts methyl sulfone in a class of chemotherapeutic agents called “Differentiation Drugs” which includes the vitamin A-related retinoid family [20], [21].

### Methyl Sulfone and Senescence

Most normal cells *in vivo* will become senescent through out the life time of an individual. For example, *in vivo*, mature melanocytes at the epidermis become senescent and remain functional for years [22], [23]. In contrast, Ben-Porath et al. suggested that a defining property of cancer cells is their inability to become senescent [24]. These authors further suggest that senescence is “a physiological response of normal cells that must be overcome in order for tumor development *in vivo* or cell immortalization in culture”. We show here that methyl sulfone induced approximately 99% of metastatic melanoma cells into a state of senescence. While methyl sulfone-induced senescent cells could no longer re-enter the cell cycle, these cells remained viable in culture for at least three months while retaining normal phenotypes such as arborization and production of melanin. Our data suggest that triggering cancer cells into a senescent state with methyl sulfone may be an effective approach to subdue the lethal properties of metastatic melanoma. The concept of induction of senescence rather than apoptosis of cancer cells as an approach to control cancer was first proposed by Serrano et al. in 1997 [25]. Over the last decade or so, the concept of chemotherapy-induced senescence has generated increasing interest [24], [26], [27], [28], [29].

### Conclusion

These studies demonstrate that changing the microenvironment by addition of this molecule changes the life history of metastatic Cloudman M3 melanoma. The possibility that metastatic melanoma cells can be “re-programmed” suggests a powerful tool that permits us to drill down more precisely into mechanisms that lead to the emergence of cancer pathology.

In conclusion, methyl sulfone may be important, first, as an effective and non-toxic chemotherapeutic compound to treat metastatic melanoma cells and perhaps other metastatic cancers. Additionally, methyl sulfone appears to reprogram metastatic Cloudman M3 melanoma cells into normal healthy melanocytes. From these studies, we speculate that methyl sulfone-induced differentiation may circumvent the development of resistance in healthy melanocytes. Of equal importance, these studies suggest that methyl sulfone may be a tool for basic scientists to decipher fundamental processes whose mechanisms are not well understood: what induces or reverses contact inhibition; what regulates induction of senescence; what signaling pathways play a role in the transformation of healthy cells to cancer cells and back to healthy cells.

## Supporting Information

Movie S1 Live cell video of confluent melanoma cells incubated in RPMI medium with 200 mM methyl sulfone.
(0.91 MB MPG)

Movie S2 Live cell video of melanoma cells in RPMI medium in the absence of methyl sulfone.
(0.32 MB MPG)
